# Comparison of Prescribed Physical Therapy to a Home Exercise Program for Pediatric Sports-Related Concussion Patients

**DOI:** 10.3390/children9091371

**Published:** 2022-09-10

**Authors:** August M. Price, Nicholas Arvin, Benjamin Seagraves, Scott O. Burkhart, Gregory Knell

**Affiliations:** 1 Children’s Health Andrews Institute, Plano, TX 75024, USA; 2 Department of Psychiatry, University of Texas Southwestern, Dallas, TX 75390, USA; 3 Bellapianta Orthopaedics & Sports Medicine, Montclair, NJ 07042, USA; 4 Children’s Health Center for Youth Sport, Activity, and Health, Southern Methodist University, Dallas, TX 75205, USA; 5 Department of Epidemiology, Human Genetics, and Environmental Sciences, The University of Texas Health Science Center at Houston, Houston, TX 77030, USA; 6 Center for Pediatric Population Health, Children’s Health, The University of Texas Health Science Center at Houston, Dallas, TX 75390, USA

**Keywords:** concussion, physical therapy, neurorehabilitation, pediatric, protracted recovery

## Abstract

The purpose of this retrospective chart review was to compare sports-related concussion (SRC) recovery time in protracted recovery (≥28 days) patients who were prescribed physical therapy (PPT) with those who were only provided a home exercise program (HEP). We hypothesized PPT would be associated with shorter recovery times relative to HEP. Associations were evaluated with multivariable zero-truncated negative binomial regressions. Among the 48 (30.2%) PPT and 111 (69.8%) HEP patients, the majority were female (57.9%), the mean age was 15.3 ± 1.4 (PPT) and 14.2 ± 2.8 (HEP), and time to clinic was a median 6.0 (IQR = 3.0–27.0; PPT) and 7.0 (IQR = 3.0–23.0; HEP) days. After adjusting for demographic (age, sex) and clinical measures (concussion history, convergence, VOMS, PCSS score, and days to clinic), PPT unexpectedly was associated with 1.21 (95% CI: 1.05, 1.41) additional recovery days compared with HEP. One reason for this could be related to patients adhering to the number of a priori prescribed PT sessions which may or may not have aligned with the patient’s symptom resolution. Future research should explore this hypothesis while aiming to evaluate the effect of PPT versus HEP using a randomized design. If confirmed, these findings are encouraging for patients who could not otherwise access or afford specialty rehabilitation.

## 1. Introduction

Public health concerns have grown regarding the short- and long-term consequences of sports-related concussion (SRC) in youth athletes, resulting in the development of protocols designed to improve the management of this injury [1]. Although many athletes seek treatment from primary care providers, professional consensus groups recognize that SRC is best managed by a collaborative or multidisciplinary team, including but not limited to physicians, neuropsychologists, physical/vestibular/vision therapists, behavioral health counselors, and certified athletic trainers [2,3,4]. This is in large part due to the heterogeneous nature of SRC recovery which may include vestibular and ocular motor dysfunction [5,6,7], neurocognitive deficits [8,9], posttraumatic migraine [10], sleep disruption [11], cervicogenic injury [12,13], and anxiety [14].

A primary goal of pediatric SRC management is to utilize treatments that reduce recovery time, which is ultimately defined as returning to normal activity, including returning to full sport participation for youth athletes. Previous work has attempted to identify rehabilitation factors that may contribute to a reduction in recovery time. Four randomized clinical trials examined early sub-symptom threshold aerobic exertional effects on concussion recovery intervals; two showed promise [15,16], one found no benefits [17], and one study showed trends toward reduced symptom burden but prolonged medical clearance [18]. Other groups have shown vestibular/ocular motor rehabilitation for SRC to be a promising tool with respect to improving symptoms, visuo-vestibular performance, and reducing recovery intervals [19,20,21]. To rule out vestibular and/or ocular motor involvement, the Vestibular/Ocular Motor Screening (VOMS) tool has been validated to measure unique aspects of vestibular and ocular motor functions not captured by other traditional assessments [22]. Utilizing specific gaze stability and oculomotor control exercises, vestibular rehabilitation may improve VOMS performances in pediatric patients with concussion [23]. However, access to licensed therapists specializing in vestibular rehabilitation is generally limited to larger metropolitan areas and specialty clinics.

In place of specialty vestibular therapy clinics, physical therapy (PT) has emerged as a potentially more accessible rehabilitation option for SRC patients that provides unique expertise in assessing physiological readiness to return to activity and the ability to facilitate components of a vestibular/ocular motor rehabilitation. Physical therapy for concussion involves a multifaceted approach reviewing musculoskeletal, oculomotor, vestibular, and aerobic systems to determine the best course of treatment, which typically includes a combination of vestibular/ocular motor rehabilitation and sub-symptom threshold aerobic exercise. In April 2020, the Clinical Practice Guidelines for Physical Therapy Evaluation and Treatment After Concussion/Mild Traumatic Brain Injury published guidelines on PT as a primary intervention for SRC management for adults and outlined recommendations for the screening/diagnosis and examination/intervention of cervical musculoskeletal, vestibulo-ocular, autonomic/exertional, motor, and psychosocial co-morbidities associated with concussion [24].

What is not yet known is whether a home-based vestibular/ocular motor rehabilitation program could be useful for patients who could not otherwise access or afford specialty rehabilitation services. Thus, the purpose of this retrospective chart review was to explore the differences in recovery time between those prescribed PT versus those provided with an unsupervised home-based exercise program (HEP) among a sample of pediatric SRC patients with protracted recovery. We chose to investigate patients with protracted recovery (≥28-days of recovery) because these patients are more often referred to specialty rehabilitation clinics such as physical and/or vestibular therapy [25]. We hypothesized that patients referred to PT would benefit from the structure of provider facilitated rehabilitation and consequently experience shorter recovery times compared with patients instructed to complete HEPs.

## 2. Materials and Methods

### 2.1. Study Design

This study was a retrospective chart review using data from electronic medical records (EMR) of patients who presented to an outpatient specialty sports medicine clinic from October 2018 to October 2021 for a suspected concussion. All patients were followed by clinic staff (at approximately one-to-two-week intervals) from the date of initial visit until the date of medical clearance. Data collected from the EMR included SRC diagnosis (yes/no), age (years), gender (male or female), injury date (month, day, year), date of medical clearance (month, day, year), referral to PT (yes/no), total PT visits (count), VOMS performance (sum of differences in symptom provocation scores from baseline for each VOMS test). Date of injury was self-reported, and date of medical clearance was determined by a licensed healthcare professional and included symptom tracking with the Post-Concussive Symptom Scale (PCSS), the VOMS, and neurocognitive testing.

The sampling method employed was convenience based in that eligible patients were selected over the study observation based on their available data. Eligibility criteria included patients between ages 13–18 who were diagnosed with a SRC based on criteria put forth by the Concussion in Sport Group [3], and endured a protracted recovery (≥28 days). In an effort to maintain generalizability, we included patients with initial evaluations before and after the 28-day mark. Exclusion criteria included patients who were referred to PT but did not initiate therapy or did not complete at least three PT sessions. Three PT sessions was chosen as the cut off to ensure this modality received adequate exposure to the study treatment. Additional exclusion criteria included prominent cervicogenic injuries, history of neurodevelopmental disorder other than ADHD and/or a specific learning disability and congenital or acquired neurological disorders not related to concussion. This retrospective chart review was approved on 05 July 2021 by the local institutional review board (STU-2021-0334) and deemed exempt from requiring informed consent.

### 2.2. Primary Exposures of Interest

**Prescribed physical therapy (PPT)** was completed with a licensed physical therapist trained in concussion rehabilitation and the referral was made at the time of the initial evaluation. As such, some patients initiated PPT after the 28-day mark. Patients were referred to physical therapy at the discretion of the healthcare professional and based on an initial assessment of vestibular and ocular motor dysfunction. Headache and cervical injury symptoms often overlap [12], thus, all PPT patients received a comprehensive physical examination including a cervical passive/active range of motion (ROM) assessment to rule out spinal involvement, and an assessment for muscle trigger points through cervical and thoracic regions [26]. If decreased cervical ROM was present, manual interventions such as High Velocity Low Amplitude Thrust spinal manipulation was used to improve cervical ROM while reducing cervical strain [27,28]. Then, the PPT patients completed vestibular/ocular motor rehabilitation (e.g., moving target pursuits, horizontal and vertical saccades, and vestibulo-ocular reflex [VOR] and visual motion sensitivity [VMS]). Patients performed graduated sub-symptom threshold exertion training (e.g., walking/light jogging on a treadmill and riding on a stationary bike) at the discretion of the physical therapist and based on established guidelines [24], one to two times per week, and were further instructed to complete the vestibular/ocular motor exercises at home once per day. Heart rate monitoring was not included as part of this PT protocol.

**Home-exercise program (HEP)** patients were any of those who either could not initiate PT (e.g., due to financial limitations) or were not deemed appropriate for PT at the discretion of the healthcare professional. HEP patients were provided with vestibular/ocular motor rehabilitation protocols, developed by a physical therapist with concussion expertise, and instructed to complete each exercise three times per day. A certified athletic trainer provided each patient with the HEP at the time of their initial visit and demonstrated how to complete each exercise. Patients were asked to complete the HEP protocol once in front of the athletic trainer and were instructed to correct errors if necessary. The exercise protocol took approximately 15-min to complete and included moving target smooth pursuits, timed horizontal and vertical saccades, “pencil pushups” (near-point convergence retraining), “head nods” (VOR activation), and VOR cancellation.

All patients were provided with standard return-to-learn and return-to-play protocols, concussion-specific aerobic exercise recommendations, behavioral health recommendations (e.g., sleep hygiene, hydration, and diet), and psychoeducation on the effects and expected recovery trajectory of SRC.

### 2.3. Primary Outcome of Interest

The primary outcome of interest was recovery time in days. Recovery time was defined as the number of days from date of injury to date of medical clearance. Medical clearance was determined by a licensed professional (physician, neuropsychologist, or nurse practitioner), and was defined as the patient’s date of return to unrestricted physical activity and academics participation. This included continuous full days of school without limitations or accommodations and tolerance of physical activity at the level the athlete previously endured (e.g., full contact practice without limitations). If an athlete was out of season when they recovered, they were put through a progressive exertional protocol with a physical therapist in the clinic until they were completely asymptomatic.

### 2.4. Statistical Analysis

All variables of interest used in the univariate analyses and subsequent models to evaluate the association between PPT and recovery time from a SRC compared with HEP were assessed for missing data and normality when appropriate. Patient characteristics were evaluated using frequencies and percentages, means and standard deviations (SD), and medians and interquartile ranges (IQR), depending on the characteristic’s variables’ distribution. Appropriate tests for differences in frequencies (chi-squared), means (independent samples *t*-tests), and medians (Mann–Whitney U tests) between the PPT and HEP patients were applied. To evaluate differences in SRC recovery time based on PPT treatment, the outcome of interest was a count of the number of days to recover, which is inherently absent of zeros. To account for this data structure, zero-truncated negative binomial regression models were built to estimate the relation between recovery time and PPT with the HEP group serving as the referent. The models included a crude (unadjusted) model, age-adjusted model, and a fully adjusted model. To account for the possibility of a more severe concussion among the PPT patients affecting the results, we built a multivariable model to account for initial injury severity. Variables in the fully adjusted model were also based on our previous work in this area [29,30,31], and included age, sex, concussion history (yes/no), vestibular-ocular motor dysfunction, near-point convergence, time in days between initial injury and evaluation in clinic (“days to clinic”), and PCSS score. Tests for collinearity between variables were performed along with post hoc analyses of model fit and tests for the appropriateness of the zero-truncated negative binomial model selection.

## 3. Results

This study consisted of a total of 159 eligible patients, of which 48 (30.2%) were prescribed physical therapy (PPT) and 111 (69.8%) were provided with HEPs. The mean ages for each group were 15.3 (SD = 1.4) for PPT patients and 14.2 (SD = 2.8) for HEP patients. The majority of HEP patients were female (60.4%), while PPT patients were more evenly split in terms of sex (52.1% female). Across both groups, most of the patients were white race/ethnicity, including 66.7% for HEP and 68.8% for PPT patients. A similar history of concussion was observed across the groups as well, 36.9% of HEP patients and 39.6% of PPT patients self-reported at least one previous concussion. The median (interquartile range [IQR]) time to clinic for PPT patients was 6.0 (IQR = 3.0–27.0) days and 7.0 (IQR = 3.0–22.0) days for the HEP patients. Overall recovery time was longer for the PPT patients with a median 52.0 (IQR = 35.0–83.5) days, compared with a median 39.0 (IQR = 33.0–57.0) days for HEP patients. Complete patient characteristics are provided in Table 1.

Table 2 provides the results of the crude and adjusted models estimating the association between PPT modality and recovery time when compared to the referent/standard treatment group (HEP). In all of the models, PPT was associated with about 1.2 days longer recovery time compared to HEP. However, only the crude model (Model 1) and the fully adjusted model (Model 3), were found to be statistically significant. For instance, the fully adjusted model’s (Model 3) results demonstrate that receiving PPT was associated with 1.21 (95% CI: 1.05, 1.41) days longer recovery time than HEP after accounting for age, sex, concussion history, VOMS dysfunction, convergence, days to clinic, and PCSS score.

## 4. Discussion

The purpose of the current study was to compare the recovery time in days between SRC patients prescribed physical therapy (PPT) and those provided with a home exercise program (HEP), all of whom experienced protracted recovery (≥28-days). Protracted recovery patients were selected given the higher likelihood that complicated patients would be referred to specialty rehabilitation clinics [25]. We hypothesized that PPT patients would experience faster recovery times compared with HEP patients due to the structure of a provider facilitating the rehabilitation program and the recommended use of PT for improving concussion outcomes in adults [24,32,33]. Unexpectedly, after controlling for factors related to injury severity and protracted recovery (e.g., sex, age, concussion history, vestibular-ocular motor dysfunction, convergence distance, days to clinic, and PCSS score), results showed that PPT was associated with 1.21 (95% CI: 1.05, 1.41) more days of recovery relative to patients completing HEPs. It is important to note that the fully adjusted model’s measure of association between treatment modality and recovery time did not differ significantly from the unadjusted or age-only adjusted models’ measures of association. This would indicate that none of the variables included in the multivariable models were significantly confounding any association between treatment modality and recovery time, including variables associated with initial injury severity. However, although the fully adjusted model’s effect size did not differ, the confidence limits did narrow, indicating a greater level of precision in the fully adjusted models. The clinical importance of this increase in precision is negligible.

We posit three potential explanations for this finding, each of which deserves further exploration. The first emerged after a post hoc review of each PPT patients’ EMR. Based on discharge dates abstracted from the record, 18.8% of PPT patients were recommended to continue PT (at least one additional visit) after achieving full symptom resolution based on symptom reporting and VOMS performance. There are several potential explanations for this discrepancy between symptom resolution and time to clearance. It may be due to an abundance of caution by the physical therapist; there may have been reason to believe the patient was not fully recovered despite reporting symptom resolution (e.g., qualitative findings on the examination that did not appear in the EMR); or patients may have been adhering to an a priori prescription of PT sessions which may or may not align with symptom resolution.

A second possible explanation for the present study’s findings may be related to selection bias due to nonrandom assignment of patients, despite our efforts to control for this by adjusting for initial injury severity in the multivariable models. The PPT patients reported significantly higher symptom burden on the PCSS during the initial visit which has been shown to be associated with prolonged recovery [34,35,36]. It is possible that the PPT patients had more complicated concussions and thus would have experienced an even longer recovery if not for PT. We attempted to minimize this potential confound by including only patients with a protracted recovery and controlling for variables known to be related to protracted recovery and injury severity including PCSS and VOMS, which were both collected at the initial visit. However, due to the observational design of the study, it is not possible to definitively conclude that PPT patients did not suffer a more complicated injury. We additionally note that initial VOMS performances between the two groups did not differ (*p* = 0.76), and the VOMS has previously been shown to be a strong predictor of protracted recovery in pediatric concussion patients [29,30,37,38,39].

Finally, these results indicate that it is worth exploring a dose–response relationship between the number of times a patient completed their vestibular/ocular motor rehabilitation and days to recovery, regardless of setting. Although we do not know how often patients completed their home exercises, each HEP patient was instructed to complete a vestibular/ocular motor rehabilitation program three times per day. In contrast, PPT patients completed structured rehabilitation, usually twice per week, that included vestibular/ocular motor rehabilitation and graded sub-threshold exertion training with a recommendation to complete their exercises at home at least once per day when they did not have a PT appointment. A primary difference between these treatment modalities might be the amount of time spent engaged in vestibular/ocular motor rehabilitation; PPT involves targeted interventions with a trained professional for a shorter increment of time per week while the home-based protocols potentially involve more doses of a titrated rehabilitation protocol each day. This final potential explanation should be interpreted cautiously as the retrospective nature of this study did not allow for tracking of HEP adherence.

Regardless of the reason for the present study’s unexpected results, the current findings provide an incentive to develop an empirically validated PT protocol for pediatric SRC patients. At present, there are differing opinions within the PT discipline regarding number of visits, length of treatment, and when to commence and discontinue treatment for concussion patients [24,33,40]. Based on our results, future protocols should be supported with research that delineates the frequency with which patients should complete vestibular/ocular motor rehabilitation, at home or in-clinic, and should additionally include definitive discontinuation criteria to avoid burdening patients with undue travel and expenses. Additionally, we would note that although 1.2 days of longer recovery for PPT patients may not appear to be a clinically relevant finding, the inverse of this relationship was expected. The finding that recovery time for patients receiving PPT was statistically similar to recovery time for those completing HEPs may be useful information for those who could not otherwise access or afford specialty rehabilitation.

This study is not without limitations. Although every effort was made to instruct patients to complete HEPs as prescribed, as is the case with any at-home treatment program, it is not known how often and to what extent each patient completed their rehabilitation, limiting our ability to draw conclusions about a dose-response relationship. Additionally, although both groups were provided the same recommendations regarding aerobic physical exercise, it is possible that the HEP treatment group engaged in more physical activity than the PPT patients, which could have contributed to faster recovery [41,42]. Finally, generalizability is limited by a relatively homogenous sample related to race and ethnicity and due to the analytic sample receiving care within a specialty concussion clinic in a major metropolitan area with onsite access to PT and healthcare professionals trained in the management of SRC. The extrapolation of these findings to other settings should be considered with caution.

## 5. Conclusions

The current study compared the recovery time of SRC patients with protracted recovery who either completed PPT or HEPs. Results showed that PPT was not associated with a faster recovery relative to patients provided with HEPs. This finding is in contrast to current guidelines recommending PT for concussion recovery and warrants further exploration. Tentatively, there may be an association between the number of times a patient completes vestibular/ocular motor rehabilitation and recovery time, though this would require further exploration using measures of treatment compliance and volume. Additionally, several PPT patients in our study may have attended at least one additional PT session than was necessary, resulting in delayed medical clearance. Future research should examine this hypothesis using a randomized controlled trial to better understand the therapeutic benefits of HEP vs. PPT.

## Figures and Tables

**Table 1:** Descriptive statistics on a sample of pediatric patients presenting to a specialty sport-related concussion clinic and subsequently experiencing a protracted recovery (≥28 days) by treatment modality, 2018–2021.

Characteristic	Overall	Treatment Modality, *n* (%)		*p*-Value
		HEP	PPT	
**Overall**	159 (100.0)	111 (100.0)	48 (100.0)	<0.0001
**Age, years**				
Years, mean (SD)	14.5 (2.5)	14.2 (2.8)	15.3 (1.4)	0.008
**Sex**				0.332
Male	67 (42.1)	44 (39.6)	23 (47.9)	
Female	92 (57.9)	67 (60.4)	25 (52.1)	
**Race/ethnicity**				0.830
White	107 (67.3)	74 (66.7)	33 (68.8)	
Black/African American	18 (11.3)	14 (12.6)	4 (8.3)	
Hispanic/Latino	6 (3.8)	3 (2.7)	3 (6.3)	
Other	28 (17.6)	20 (18.0)	8 (16.7)	
**History of concussion**				0.752
No	99 (62.3)	70 (63.1)	29 (60.4)	
Yes	60 (37.7)	41 (36.9)	19 (39.6)	
**VOMS ^a^**				
Sum of differences, median (IQR)	16.0 (6.0–32.5)	16.0 (5.5–34.0)	17.0 (9.5–31.5)	0.760
**Convergence**				
Distance, median (IQR)	4.7 (1.0–10.7)	5.0 (1.0–10.7)	4.5 (1.0–10.0)	0.919
**PCSS**				
Total, median (IQR)	36.0 (16.0–60.0)	25.5 (14.0–53.0)	50.0 (24.0–73.0)	0.010
**Recovery time**				
Days, median (IQR)	42.0 (33.0–65.0)	39.0 (33.0–57.0)	52.0 (35.0–83.5)	0.008
**Time to clinic**				
Days, median (IQR)	7.0 (3.0–23.0)	7.0 (3.0–22.0)	6.0 (3.0–27.0)	0.810

Abbreviation: PPT, prescribed physical therapy; HEP, home exercise program; IQR, interquartile range; SD, standard deviation; VOMS, Vestibular/Ocular Motor Screening; PCSS, Post-Concussion Symptom Scale. Notes: ^a^ Computed as the sum of differences in symptom scores from baseline to post-VOMS symptom provocation testing.

**Table 2:** Models estimating the association between PPT concussion treatment modality and recovery time among sample of pediatric patients presenting to a specialty sport-related concussion clinic and subsequently experiencing a protracted recovery (≥28 days), 2018–2021.

	Standardized Coefficients			Unstandardized Coefficients			
Models	β	SE	95% CI	IRR	SE	95% CI	*p*
Model 1	0.25	0.11	0.04, 0.45	1.28	0.13	1.04, 1.58	0.02
Model 2	0.20	0.11	−0.01, 0.41	1.22	0.13	0.98, 1.51	0.07
Model 3	0.19	0.07	0.05, 0.34	1.21	0.09	1.05, 1.41	0.009

Abbreviation: PPT, prescribed physical therapy. Model 1: crude model relating PPT treatment modality versus home exercise program to recovery time (days). Model 2: Model 1 + age adjustment. Model 3: Model 2 + sex, concussion history, vestibular-ocular motor dysfunction, days to clinic, PCSS score, and near-point convergence adjustment.

## Data Availability

The data presented in this study are available on request from the corresponding author.
